# Supplementary material for: Characterization of an α-Amylase from the Honeybee Chalk Brood Pathogen Ascosphaera apis
Source: J Fungi (Basel). 2023 Nov 5;9(11):1082. doi: 10.3390/jof9111082 (PMC10672707; doi:10.3390/jof9111082)
Supplement: Supplementary file 1 [file jof-09-01082-s001.zip › Supplementary_Material 20231101.pdf]

Supplementary Material

Characterization of an α-amylase from the honey bee chalk brood pathogen *Ascosphaera apis*

Jincheng Li, Sen Li, Chenjie Yang, Nemat O. Keyhani, Huili Pu, Longbin Lin, Xiaoxia Li, Peisong Jia, Dongmei Wu, Jieming Pan, Philip C Stevenson, G Mandela Fernández-Grandon, Liaoyuan Zhang, Yuxi Chen, Xiayu Guan*, Junzhi Qiu*

Correspondence: Xiayu Guan: 000q021008@fafu.edu.cn; Junzhi Qiu: 000q120055@fafu.edu.cn.

1 Supplementary Data

1.1 Supplementary Tables

Table S1. Plackett-Burman design matrix and results of screening of variables affecting amylase activity by *A. apis*

Run	X_1	X_2	X_3	X_4	X_5	X_6	X_7	X_8	Amylase activity (U/mL)
1	1	-1	1	-1	-1	-1	1	1	30.95 (1.47)
2	1	1	-1	1	-1	-1	-1	1	34.60 (2.01)
3	-1	1	1	-1	1	-1	-1	-1	30.32 (1.58)
4	1	-1	1	1	-1	1	-1	-1	37.92 (0.83)
5	1	1	-1	1	1	-1	1	-1	35.69 (0.72)
6	1	1	1	-1	1	1	-1	1	30.24 (0.96)
7	-1	1	1	1	-1	1	1	-1	32.78 (1.15)
8	-1	-1	1	1	1	-1	1	1	30.42 (1.17)
9	-1	-1	-1	1	1	1	-1	1	32.85 (2.19)
10	1	-1	-1	-1	1	1	1	-1	33.94 (1.61)
11	-1	1	-1	-1	-1	1	1	1	24.97 (1.04)
12	-1	-1	-1	-1	-1	-1	-1	-1	35.93 (1.82)

X_3 and X_6 for amylase activity were dummy factors. Other codes were the variables of the medium to be optimized. X_1, X_2, X_4, X_5, X_7 and X_8 were maltose, yeast powder, $CaCl_2$, $ZnCl_2$, vitamin E and pH, respectively. Values for amylase are means (SE) of three replicate flasks.

Table S2. The range and the level of variables independently tested in BBD for *A. apis* amylase activity

Code	Variable for amylase activity	Level(g/L)		
		-1	0	1
X_1	Maltose	45.5	46	46.5
X_2	$CaCl_2$	1.44	1.49	1.54
X_3	pH	6.4	6.6	6.8

Table S3. The matrix of Box–Behnken design for optimization of *A. apis* amylase activity. Values for amylase activity are means (SE) of three replicate flasks

Run	X_1	X_2	X_3	Amylase activity (U/mL)
1	-1	-1	0	39.64 (2.12)
2	-1	1	0	37.15 (1.68)
3	1	-1	0	32.10 (1.43)
4	1	1	0	44.05 (2.27)
5	0	-1	-1	33.10 (1.54)
6	0	-1	1	42.13 (1.79)
7	0	1	-1	42.09 (1.56)
8	0	1	1	36.03 (2.11)
9	-1	0	-1	42.49 (2.05)
10	1	0	-1	33.37 (1.42)
11	-1	0	1	35.05 (1.87)
12	1	0	1	37.09 (1.63)
13	0	0	0	46.15 (2.04)
14	0	0	0	46.10 (1.91)
15	0	0	0	45.71 (2.18)

Table S4. ANOVA for response surface quadratic model of amylase activity

Source	DF	SS	MS	F	Pr > F
Model	9	329.6894	36.63216	11.22671	0.0080
Error	5	16.31473	3.262947		
(Lack of fit)	3	16.20041	5.400137	94.47234	0.0105
(Pure Error)	2	0.114322	0.057161		
Total	14	346.0042			
R^2	0.9528	Adjusted R^2	0.8680		

1.2 Supplementary Figure

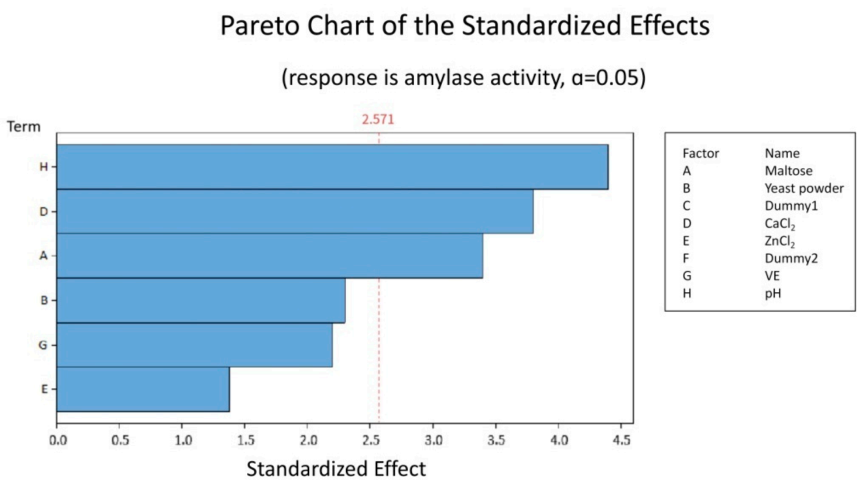

Figure S1 Pareto chart of the standardized effects for amylase activity.
